# Search for non-lactam inhibitors of mtb β-lactamase led to its open shape in apo state: new concept for antibiotic design

**DOI:** 10.1038/s41598-017-06023-3

**Published:** 2017-07-24

**Authors:** Amin Sagar, Nazia Haleem, Yaawar Mir Bashir, Ashish

**Affiliations:** CSIR-Institute of Microbial Technology, Chandigarh, India

## Abstract

Mtb β-lactamase (BlaC) is extremely efficient in hydrolyzing ß-lactam antibiotics which renders/leads to protection and/or resistance to this bug. There is a compelling need to develop new non-lactam inhibitors which can bind and inhibit BlaC, but cannot be hydrolyzed, thus neutralizing this survival mechanism of Mtb. Using the crystal structure of BlaC we screened 750000 purchasable compounds from ZINC Database for their theoretical affinity to the enzyme’s active site. 32 of the best hits of the compounds having tetra-, tri- and thiadi-azole moiety were tested *in vitro*, and 4 efficiently inhibited the enzymatic activity of recombinant BlaC. Characterization of the shape of BlaC−/+ inhibitors by small angle X-ray scattering (SAXS) brought forth that BlaC adopts: (1) an open shape (radius of gyration of 2.3 nm compared to 1.9 nm of crystal structures) in solution; (2) closed shape similar to observed crystal structure(s) in presence of effective inhibitor; and (3) a closed shape which opens up when a hydrolysable inhibitor is present in solution. New BlaC inhibitors were: 1-(4-(pyridin-3-yl)-thiazol-2-ylamino)-2-(7,8,9-triaza-bicyclo[4.3.0]nona-1(6),2,4,8-tetraen-7-yl)-ethanone; 8-butyl-3-((5-(pyridin-2-yl)-4H-1,2,4-triazol-3-ylamino)-formyl)-8-aza-bicyclo[4.3.0]nona-1(6),2,4-triene-7,9-dione; 1-(3-((5-(5-bromo-thiophen-2-yl)-1,3,4-oxadiazol-2-yl)-methoxy)-phenyl)-1H-1,2,3,4-tetraazole; and 1-(2,3-dimethyl-phenylamino)-2-(2-(1-(2-methoxy-5-methyl-phenyl)-1H-1,2,3,4-tetraazol-5-ylsulfanyl)-acetylamino)-ethanone. The open-close shape of BlaC questions the physiological significance of the closed shape known for BlaC−/+ inhibitors and paves new path for structure aided design of novel inhibitors.

## Introduction

 ß-lactam antibiotics are the most extensively used antimicrobial compounds worldwide and are used to treat a wide variety of bacterial infections^1^. However, their use has been limited in the treatment of Tuberculosis as *Mycobacterium tuberculosis* has been shown to be intrinsically resistant of ß-lactam antibiotics^2^. One of the reasons for this resistance is the production of the enzyme ß-lactamase (BlaC) which hydrolyses the β-lactam antibiotics^3^. It has also been proposed that the lipid rich outer cell wall of *Mycobacterium tuberculosis* presents an impenetrable barrier to ß-lactam antibiotics^4^. However, it has been experimentally demonstrated that although the penetration of ß-lactam antibiotics into a *Mycobacterium* cell is slow, considering the long cell division times, the intracellular concentrations of ß-lactam antibiotics reach a level enough for bactericidal activity^3, 5^. Therefore, BlaC is the major obstacle in the efficient use of β-lactam antibiotics to treat Tuberculosis.

BlaC has an extraordinarily broad substrate specificity with the ability to hydrolyze penicillins, cephalosporins and even carbapenems like imipenem and carbapenem^6, 7^. The enzyme is endowed with this exceptional broad specificity due to changes in its sequence and structure compared to other class A ß-lactamases. Firstly, BlaC has a much larger active site which allows it to bind and hydrolyze larger substrates^7^. Secondly, it has R154A mutation, which destabilizes the Ω loop resulting in an increased activity against cephalosporins^7^. The enzyme is also able to reverse the inhibition caused by FDA approved ß-lactamase inhibitors sulbactam and tazobactam^6^. It has been demonstrated using mass spectrometry that the enzyme is able to hydrolyze the acylated serine residue within 30 and 45 minutes for sulbactam and tazobactam, respectively, returning to its native functional state^6^. The only comforting prospect, perhaps, of these studies was that clavulanate could slowly, but irreversibly inhibit this enzyme. This spurred the efforts to develop the combination of ß-lactam antibiotics with clavulanate as a therapy against MDR (Multi Drug Resistant) and XDR (Extensively Drug Resistant) strains of *M*. *tuberculosis*. A conjugate of meropenem with clavulanate was developed and shown to be effective against 13 XDR strains of *M*. *tuberculosis* ^8^. However, recently it has been shown that a point mutation N132G enables BlaC to hydrolyze clavulanate and therefore, completely escape the irreversible inactivation^9^. This implies that a resistance against ß-lactam-clavulanate combinations will be quickly achieved by *M*. *tuberculosis* and this mode of therapy can’t be relied upon as a long term anti-tuberculosis strategy.

The three most commonly used ß-lactamase inhibitors *viz*. Clavulanic acid, Sulbactam and Tazobactam are themselves ß-lactam compounds and are therefore hydrolyzed by ß-lactamase. In addition to ß-lactamase resistance mechanism, bacteria can develop resistance to these inhibitors by mutations in porin channel(s)^10^ and having feedback sensor proteins which upregulate the levels of ß-lactamase^11^. Considering these aspects, it is logical that non-lactam ß-lactamase inhibitors would present a tougher challenge for the bacteria as they would have to evolve novel resistance mechanisms rather than adapting the existing ones. Being non-lactam, these compounds wouldn’t be hydrolysed by ß-lactamase. In addition, it is very likely that their action would not be hampered by the mutations in the porin channels and they wouldn’t trigger the ß-lactam sensing proteins to increase the expression of ß-lactamases. This thought led to the development of Avibactam, a Diazabicyclooctane compound, which was approved by FDA in February 2016 as the first non-lactam ß-lactamase inhibitor to be used to treat complex intra-abdominal infections in combination with ceftazidine and metrodinazole. Another chemically similar compound, MK-7655 has been shown to be effective against the drug resistant strains of *Enterobacteriaceae* and *Pseudomonas aeruginosa* in combination with imipenem^12, 13^. In September 2014, FDA designated MK-7655 with fast track status and participants are being recruited for Phase 3 trials. Boronates form another class of molecules which are being actively developed as non-lactam ß-lactamase inhibitors^14, 15^. RPX-7009, a boronate, was also given fast track status in April 2016 and participants are being recruited for the Phase 3 trials for testing its combination with meropenem in cases of urinary tract infection, Pyelonephritis, Pneumonia and Bacteremia. Cyclobutanone^16^ and Penam sulphate^17, 18^ inhibitors are also being developed, but are yet to reach clinical trials. As pointed out by Powers *et al*., the inhibitors which covalently bind to the activated serine residue can also bind to other active serine containing enzymes, limiting their specificity^19^. A non-lactam non-covalent inhibitor of ß-lactamase would therefore be an excellent candidate to be developed for therapeutic application(s). In this study, we used crystal structure of Mtb BlaC and virtual ligand screening protocol to identify new molecules which showed good theoretical affinity with the active site of BlaC. Finally, four new molecules were experimentally found to inhibit recombinant BlaC. While characterizing the shape of the BlaC in solution in absence and presence of inhibitors, we stumbled upon an observation that apo protein is more open than its shape understood from crystal structures, and non-hydrolyzable inhibitors keep the enzyme in closed shape.

## Results

### The crystal structures suggest a consistent conformation of BlaC

**Figure 1:**
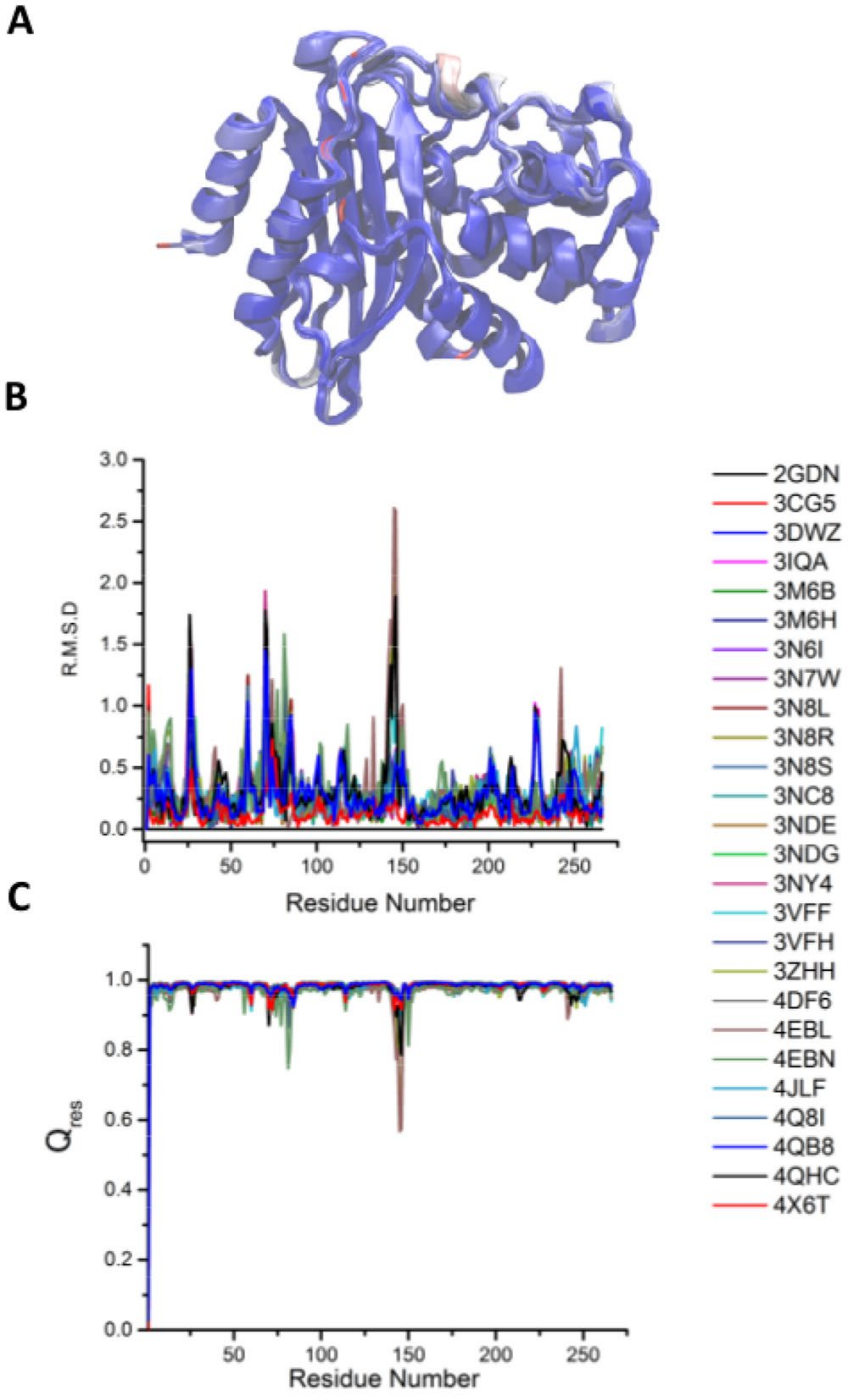
Comparison of the crystal structures of Mycobacterial ß-lactamase. (**A**) 26 crystal structures of BlaC aligned on 2GDN and colored according to their Root Mean Square Deviation (RMSD) from blue for the regions with lowest RMSD to red for the regions with highest RMSD. (**B**) and (**C**) are plots of RMSD and Q_res_, respectively.

The first step in a virtual ligand screening study is to find a reliable high resolution structure to be used as receptor/target. There are about 30 crystal structures of BlaC available in RCSB in complex with various substrates and inhibitors. In order to choose a structure for *in*-*silico* compound screening, we compared all the available structures of BlaC. It is known, that in the case of flexible proteins, the docking simulations can provide unreliable answers and/or miss the binders if the flexibility is not properly considered^20^. Therefore, we superimposed all the crystal structures of BlaC, calculated the per-residue Root Mean Square Deviation (RMSD) and Q_res_ values^21^. We expected the most flexible residues to be refined in slightly different conformations in different crystal structures and hence have a higher RMSD and lower Q_res_, but found that despite being solved in quite different buffer conditions and with a variety of substrates/inhibitors, all the crystal structures of BlaC were very similar with most regions having an RMSD of <1 Å and Q_res_ > 0.8. The aligned structures, with the residues coloured by RMSD, are presented in Fig. 1A. There were three regions, where the RMSD values were comparatively higher. These regions consist of residues 26–29, 70–85 and 143–150 (Fig. 1B). The same trend was seen in the values of Q_res_ (Fig. 1C). (It should be noted here that the residues have been numbered according to the first residue seen in most of the crystal structures *i*.*e*. the 29^th^ residue in the sequence of ß-lactamase has been numbered as the first residue). As all the structures were very similar with RMSD values for even the most flexible regions being quite low (and the Q_res_ values quite high), we concluded that any of the crystal structures would serve well as a starting structure for virtual ligand screening and proceeded with using the crystal structure of BlaC without any ligand in active site *i*.*e*. PDB ID: 2GDN for all the docking calculations.

### In-silico screening and chemical clustering of the compounds

**Figure 2:**
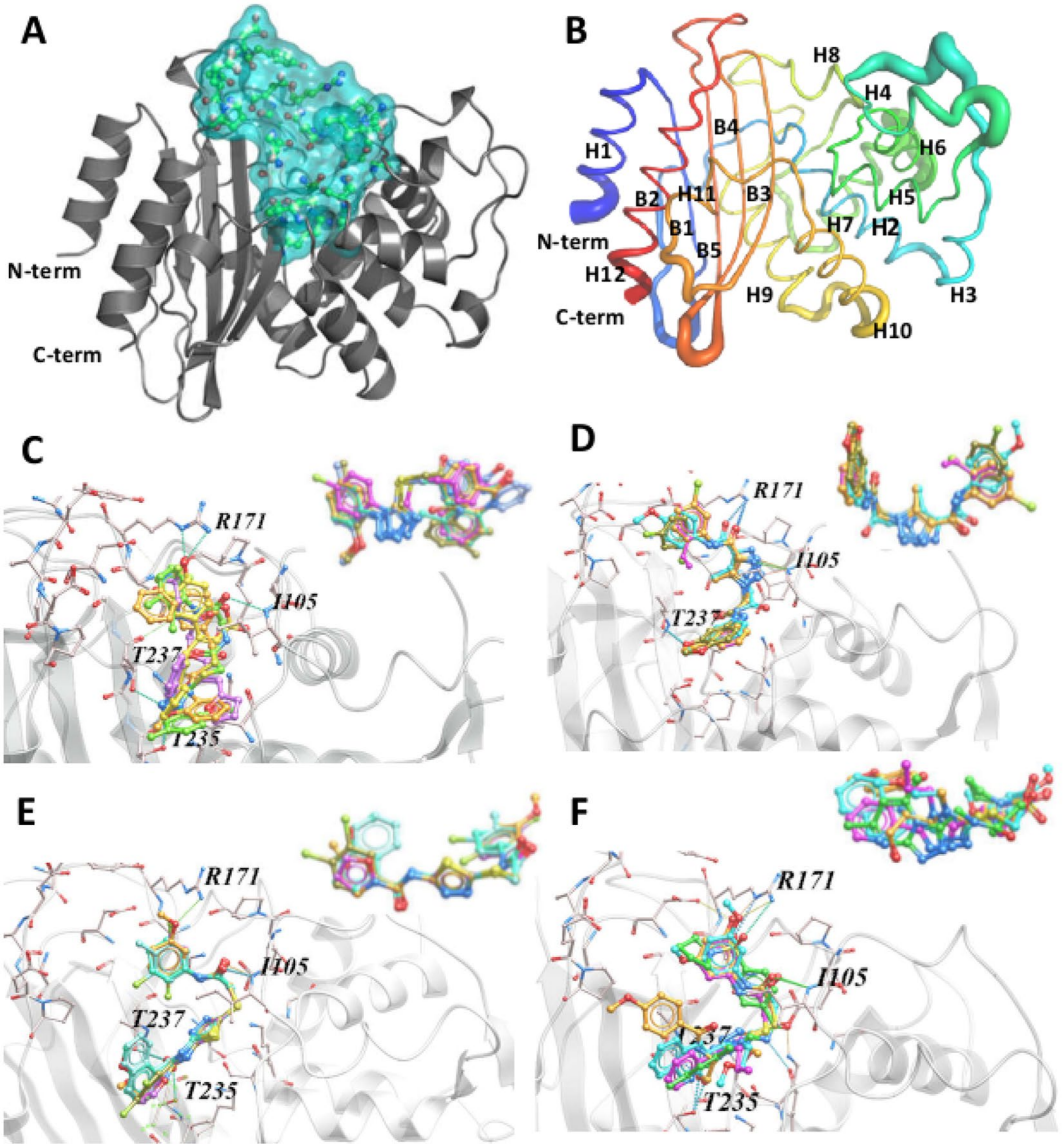
Virtual ligand screening and chemical clustering of compounds. (**A**) The crystal structure of BlaC with the residues defined as receptor displayed as sticks and the pocket formed by them displayed as cyan surface. (**B**) The crystal structure 2GDN in putty representation with the thickness depicting the B-factor and colored from blue (N terminus) to red (C terminus). The secondary structure features have been labelled with H1 through H12 representing the α-helices and B1 through B4 representing the β-sheets. (**C**–**F**) The highest scoring compounds of the four main chemical families identified by virtual ligand screening *i*.*e*. tetrazoles, 1-2-3-triazoles, thiadiazoles and 1,3,4-triazoles docked into the active site of ß-lactamase respectively. The inset shows the overlay of the compounds to emphasize the similarity in the docking poses of the compounds belonging to one family.

The crystal structure of BlaC was probed to find druggable pockets using the ICM’s Pocket finder. Interestingly, the program found only one pocket to be fitting for targeting drugs based on volume and surface area and this pocket encompassed the active site of the enzyme (Fig. 2A). B-factor putty of the C^α^ trace of the same crystal structure which reflects the local flexibility in the refined crystal structure confirmed that the coordinates around the active site were reliable (Fig. 2B). The pocket had a volume of 327.8 Å^3^, surface area of 321.1 Å^2^, a non-sphericity value of 1.39 and was composed of the residues S70, K73, S104, I105, S130, G132, E166, P167, N170, R171, T216, K234-D240 and D273-P276. We docked ~750,000 purchasable compounds from the ZINC database to the pocket described above. Ligands were optimized before calculating interactions of their different poses inside the pocket. 1135 compounds which had an ICM score better than −32 (i.e. < −32) were clustered according to the chemical groups present in them. We found that 169 of the compounds had either a thiadiazole (54) or 1,2,4-triazole (54) or tetrazole (45) or 1,2,3-triazole (16) group in them. We then compared the binding poses of the compounds having the same group and found that these compounds adopted a similar docking pose with the defining chemical groups occupying nearly identical spatial position and made similar interactions with the protein (Fig. 2C–F).

### BlaC adopts an open conformation in solution

**Figure 3:**
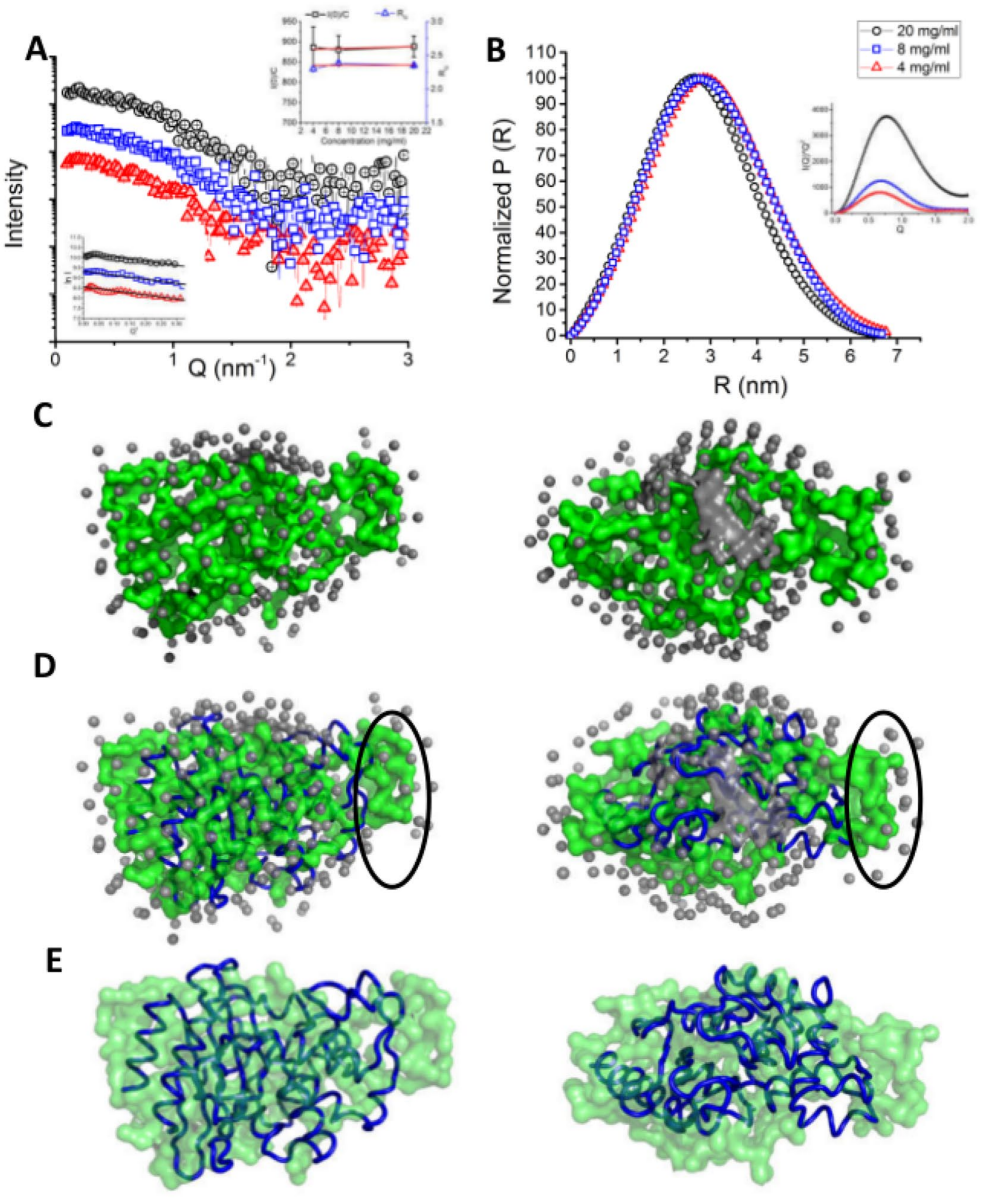
SAXS data analyses and modelling for BlaC. (**A**) SAXS intensity profiles of purified BlaC at three concentrations *i*.*e*. 4, 8 and 20 mg/ml with the Guinier plots in lower inset. The black lines show the linear fits. The upper inset has the plots of I_0_/C and R_G_ as a function of protein concentration. (**B**) The pairwise distance distribution functions (P(R)) of the SAXS datasets with the Kratky plots in inset. (**C**) Two orthogonal views of averaged dummy residue based models of BlaC. (**D**) Two orthogonal views of the crystal structure of ß-lactamase (PDB ID: 2GDN, blue tube) superimposed on the SAXS derived model. The unoccupied space is highlighted with black ovals. (**E**) Two orthogonal views of the SAXS model superimposed with the crystal structure with the water molecules hidden for better clarity.

Before doing the activity assays, we characterized the purified BlaC using SAXS data analysis to confirm that it was properly folded. However, as described below, the SAXS experiments revealed some surprising aspects of the conformational dynamics of BlaC. The SAXS intensity data was collected for BlaC at three concentrations i.e. 4, 8 and 20 mg/ml (Fig. 3A). The SAXS data showed no aggregation or inter-particle interference as there was no change in the linearity and parallel nature of the Guinier plots (ln(I(Q) vs. Q^2^) at small angles for all three concentrations (Fig. 3A lower inset). This was further confirmed by observing no significant change in the estimated intensity at zero angles (I_0_) and radius of gyration (R_G_) values as a function of BlaC concentration (from Guinier analysis, Fig. 3A upper inset). Earlier, similar conclusions have been made from SAXS data analysis for another protein^22^ and such an observation is advised before proceeding with detailed analysis^23, 24^. Additionally, the Kratky plots (I(Q)*Q^2^ vs. Q) at all the concentrations had a prominent peak which indicated that BlaC molecules possessed well folded globular shape (Fig. 3B inset). Interestingly, based on the Guinier approximation, the R_G_ was calculated to be 2.35 ± 0.012 nm (at concentration of 20 mg/ml) which was significantly larger than the value of 1.89 nm computed from the crystal structure of BlaC (PDB ID: 2GDN). Next, we calculated the distance distribution function (P(R)) by automated Indirect Fourier Transformation of the intensity profile (Fig. 3B). The distance distribution function computation considers a wider Q-range of data than Guinier approximation and provided another estimation of the R_G_ value along with the maximum linear dimension (D_max_) of the predominant shape of BlaC in solution. In correlation with Guinier approximation, the distance distribution function indicated best solution to the experimental data for a molecule with an R_G_ of 2.27 ± 0.04 nm and D_max_ of 6.7 nm which was again larger than the values expected from the crystal structure of BlaC (calculated R_G_ and D_max_ values were 1.89 and 6.1 nm, respectively using CRYSOL program and crystal structure of BlaC; PDB ID: 2GDN). Using the DATMOW program, the mass of the scattering particle was calculated to be 29.1 kDa which is in good correlation with the mass expected for the monomeric protein. These results were the first indication that BlaC adopts a more open conformation in the solution than the one seen in the crystal structures.

Using the SAXS data, the predominant solution shape of BlaC was restored using the chain-like ensemble modelling program GASBOR^25^. Using 270 dummy amino acids, ten independent models were generated and their Normalized Spatial Discrepancy (NSD) values were calculated. NSD is a quantitative measure of the similarity between two shapes with a value of 0 implying perfectly identical shapes and values >1 indicating that the shapes are systematically different^26^. The mean NSD value between the ten models was 0.954 with standard deviation of 0.015 pointing to high similarity between the independent models and therefore stability of low resolution modelling procedure. Two orthogonal views of a randomly selected model are presented in Fig. 3C. To compare the SAXS data based envelope model with crystal structure, we superimposed them by aligning their inertial axes in automated manner using SUPCOMB program (Fig. 3D and E). The SAXS model had a distinct extra volume on one side suggesting some structural rearrangement around the helix H4. Although, based solely on the SAXS data, the exact locus of structural rearrangement can’t be pinpointed and it can be argued that the crystal structure can be rotated by 180 degrees inside the SAXS envelope and C-terminal and N-terminal helices identified as centres of rearrangement, however, we favoured H4 for the following reasons. Firstly, the helix H4 has relatively long stretches of random coil regions on both sides making it flexible. Secondly, the RMSD and Q_res_ analyses identified this region as one of the most variable regions in the different crystal structures of BlaC. Moreover, this region has high B-factor in the crystal structure (as shown in Fig. 2B).

### Testing the new potential inhibitors of BlaC

**Figure 4:**
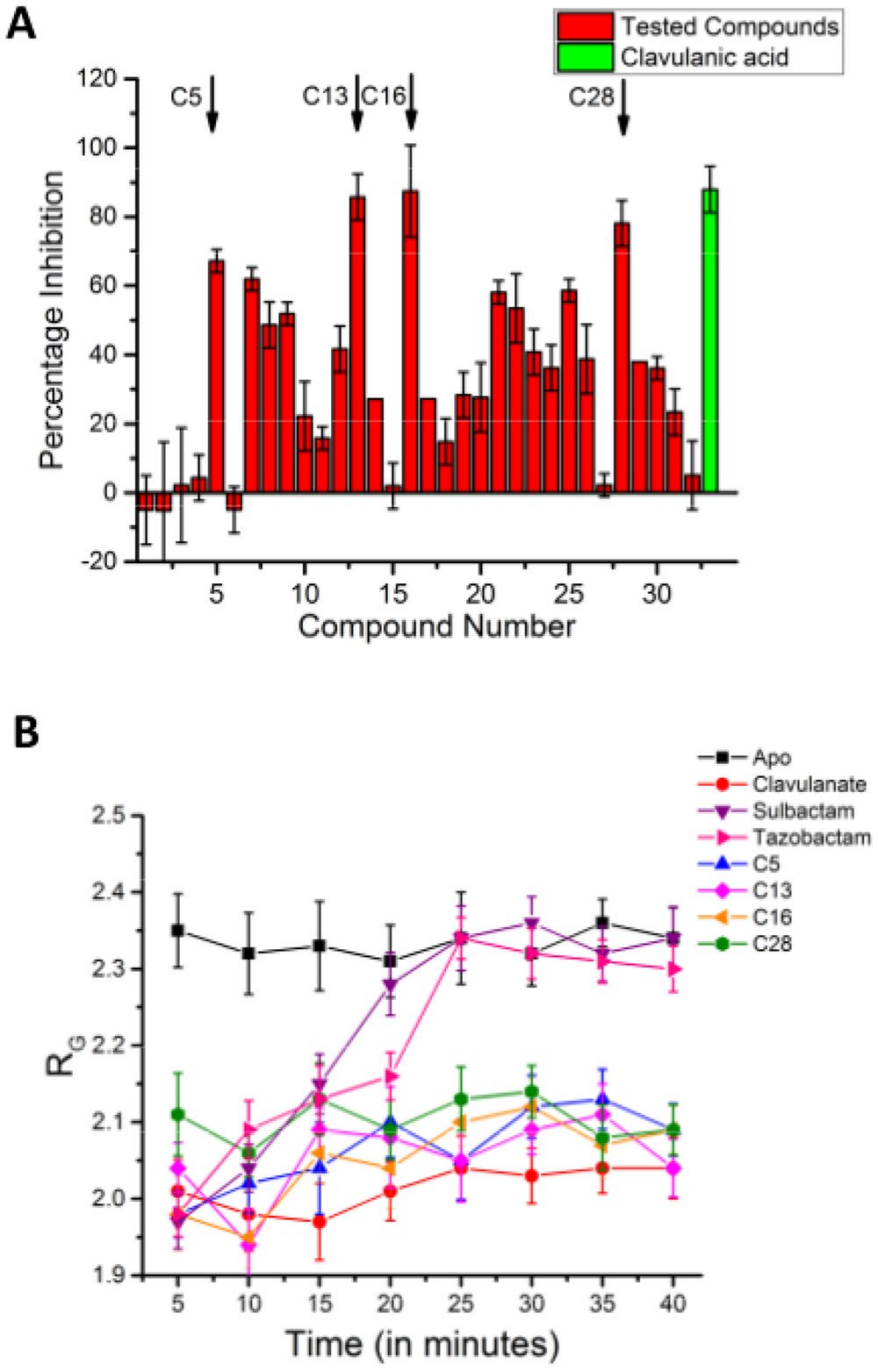
Novel inhibitors which inhibit BlaC and induce a closed conformation. (**A**) The results of the *in*-*vitro* BlaC activity assay. Three independent experiments were performed and the average values were plotted along with the standard deviation as error bars. The red bars represent the inhibitors tested (C1 through C32). The green bar represents the activity of clavulanic acid. The arrows indicate the four compounds with highest BlaC inhibitory activity (**B**) The values of R_G_ of ß-lactamase in nm plotted as a function of time in apo state and when it is incubated with ß-lactamase inhibitors sulbactam, tazobactam, clavulanic acid or the inhibitors discovered in this study (C5, C13, C16 and C28).

Recombinant BlaC was tested for its ability to hydrolyze antibiotics. We employed the well accepted protocol based on hydrolysis of CENTA, a chromogenic cephalosporin which is hydrolyzed by most β-lactamases^27^. The assay employs the simple concept that when BlaC is exposed to any molecule capable of inhibiting its activity, it will not be able to hydrolyze CENTA. Alternatively, if BlaC cannot be inhibited, it hydrolyses CENTA leading to an increase in absorbance at 405 nm due to expulsion of chromophoric entity after opening of the ß-lactam ring. 32 compounds, mentioned in Supplementary Table 1, were tested for their ability to inhibit BlaC. Percentage inhibition was mapped. As positive control, clavulanic acid was also tested which showed almost complete inhibition of BlaC in our assays. Results from average of three independent experiments are shown in Fig. 4A. Of the compounds named C1 through C32, C5, C13, C16 and C28 showed substantially high ability to inhibit BlaC. IUPAC names of these compounds are: ***C5***:1-(4-(pyridin-3-yl)-thiazol-2-ylamino)-2-(7,8,9-triaza-bicyclo[4.3.0]nona-1(6),2,4,8-tetraen-7-yl)-ethanone; ***C13***:8-butyl-3-((5-(pyridin-2-yl)-4H-1,2,4-triazol-3-ylamino)-formyl)-8-aza-bicyclo[4.3.0]nona-1(6),2,4-triene-7,9-dione; ***C16***: 1-(3-((5-(5-bromo-thiophen-2-yl)-1,3,4-oxadiazol-2-yl)-methoxy)-phenyl)-1H-1,2,3,4-tetraazole and ***C28***:1-(2,3-dimethyl-phenylamino)-2-(2-(1-(2-methoxy-5-methyl-phenyl)-1H-1,2,3,4-tetraazol-5-ylsulfanyl)-acetylamino)-ethanone.

### BlaC inhibitors induce a closed conformation in BlaC

As all the crystal structures of BlaC have been solved in the presence of its inhibitors or substrates, including structure lacking any ligand in active site i.e. PDB ID 2GDN as there was ampicillin in the mother liquor^7^, we decided to use SAXS to study if the inhibitors induce any conformational changes in BlaC. We incubated BlaC with its known inhibitors viz. clavulanic acid, sulbactam and tazobactam and also the four new inhibitors identified in this study, and collected SAXS data after every 5 minutes for 40 minutes. The SAXS profiles of BlaC alone and in the presence of various inhibitors as a function of time are presented in Supplementary Fig. S1 and the parameters used for the SAXS data collection and the calculated values are presented in Supplementary Table S2. The R_G_ of the predominant scattering species was determined as a function of time using automated Guinier approximation to track the conformation changes as the bound inhibitor gets hydrolysed (Fig. 4B). The Guinier Plots for all the SAXS intensity profiles are shown in Supplementary Fig. S2 with the comparison of the first (5 min) and last (40 min) data set in inset. Apo BlaC indicated an R_G_ value close to 2.35 nm, while in samples containing clavulanic acid, the R_G_ consistently remained around 2 nm. Not only were the R_G_ values observed with known inhibitor significantly lower than those calculated for Apo protein, but also they were closer to values calculated from the crystal structures. Interestingly, in the cases of Sulbactam and Tazobactam, the R_G_ increased gradually from 1.97 ± 0.035 nm and 1.98 ± 0.029 nm, respectively at 5 min to 2.34 ± 0.034 nm and 2.34 ± 0.027 nm, respectively at 40 minutes. The values at 5 minutes are in close agreement with the values expected from the crystal structure and the values at 40 minutes are very close to those obtained for Apo BlaC. Previously, mass spectroscopy based studies have shown that the acylated enzyme formed by sulbactam and tazobactam is hydrolysable and BlaC returns to its unmodified native state in 30–45 minutes, whereas, clavulanic acid irreversibly acylates and deactivates BlaC^6^. Finally, all our four molecules kept the R_G_ values to lower side, closer to those seen in samples containing clavulanate. Since, our molecules lacked reactive group as present in clavulanic acid, their ability to covalently bind to BlaC and thus inhibit its function was ruled out.

**Figure 5:**
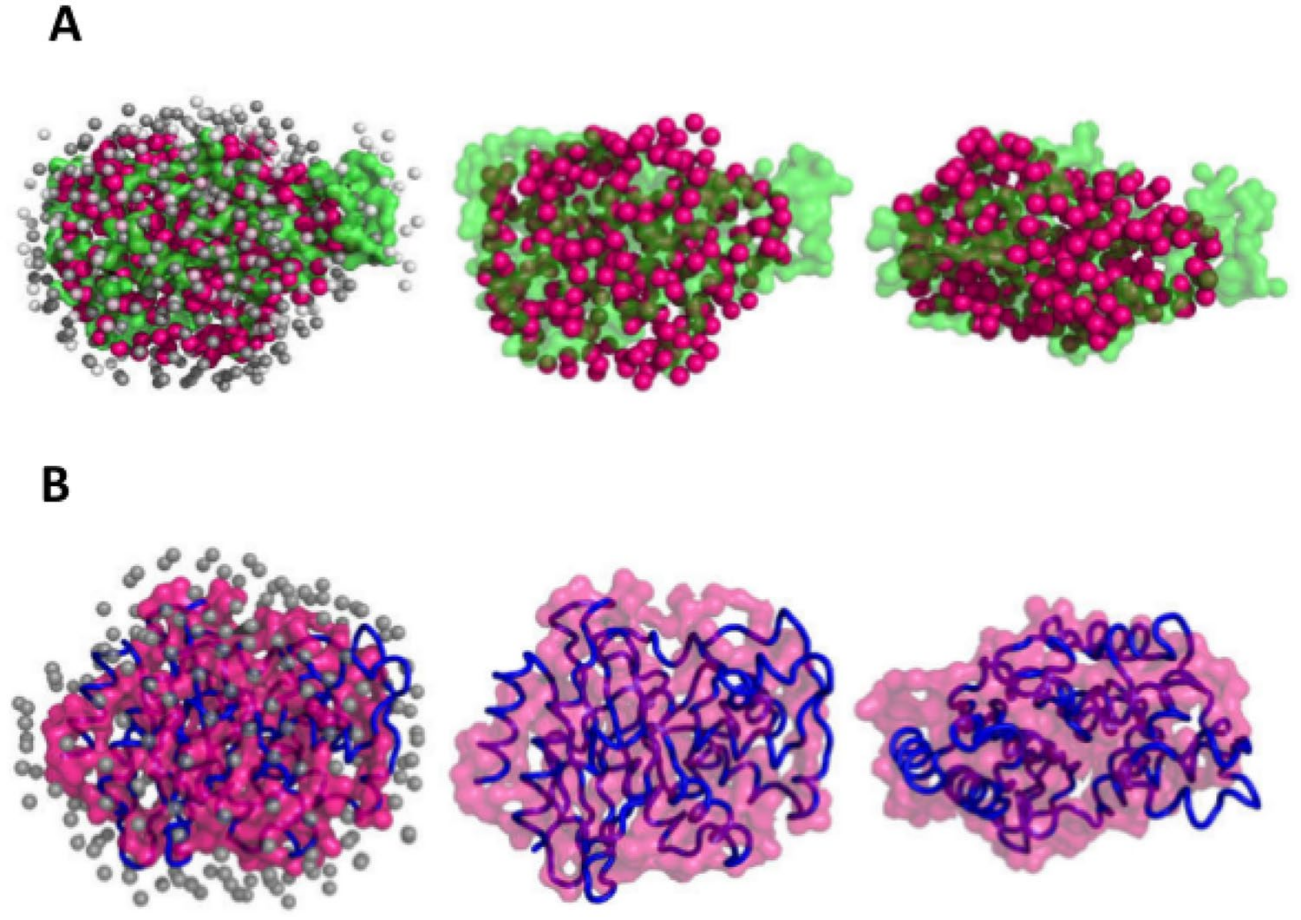
Modeling the closed shape of BlaC. (**A**) Starting from left, the SAXS model of BlaC in the presence of inhibitor C16 (Magenta) overlaid on the SAXS model of apo BlaC (green) followed by two orthogonal views of the overlaid models without the water molecules used in the structure reconstruction process, for clarity. (**B**) The SAXS model of BlaC in the presence of inhibitor C13 overlaid on the crystal structure (2GDN) followed by two orthogonal views without the water molecules (Left to Right).

The effect of the addition of various known and newly discovered inhibitors on the flexibility of BlaC was examined by Porod-Debye plots and normalized Kratky plots. In all the cases, the Porod-Debye plots showed a plateau at higher Q values and the Porod Exponent stayed between 3.7 and 4.0 (Supplementary Fig. S3). These observations suggest that BlaC behaves as a compact/folded and globular molecule with or without the presence of inhibitors^28^. This was further confirmed by the normalized Kratky plots, all of which showed a peak close of a value of 1.73 on the x-axis which is characteristic of globular proteins (Supplementary Fig. S4). SAXS profiles of the BlaC having clavulanic acid and other inhibitors were used to restore the predominant scattering shape of our BlaC in presence of these molecules (only BlaC + C16 is shown, Fig. 5). The models restored for BlaC with effective inhibitors were clearly smaller than those for Apo BlaC (Fig. 5A). Automated superimposition of the SAXS data based model of BlaC + C16 with the crystal structure of Apo BlaC showed high resemblance (Fig. 5B).

**Figure 6:**
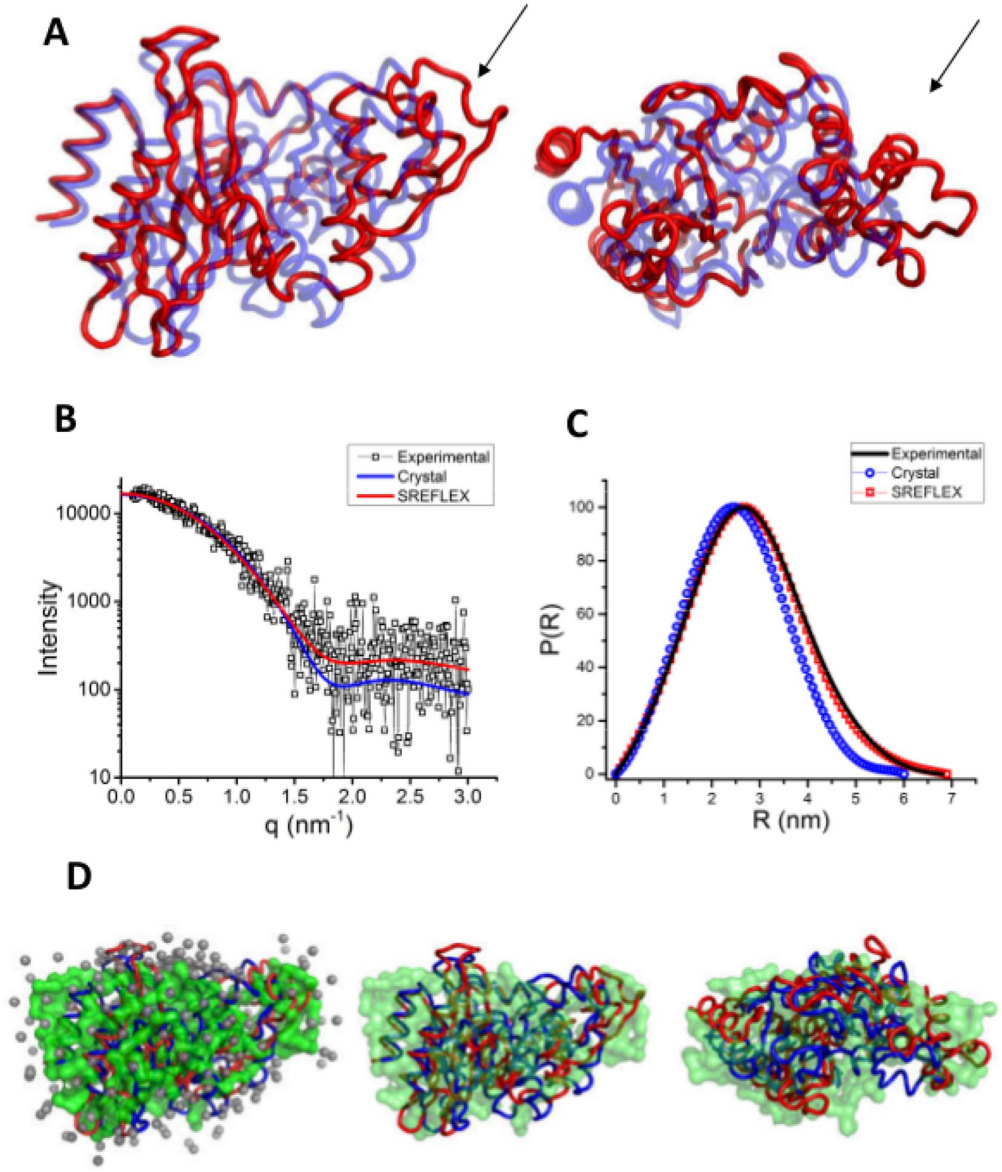
Using NMA and SAXS to derive the open model of BlaC. (**A**) The SREFLEX derived model and the crystal structure overlaid on each other. The loop showing the most prominent change is highlighted by a black arrow. (**B** and **C**) The fit between the theoretical SAXS profiles and distance distribution functions of closed form of BlaC, as seen in the crystal structures, and the open form derived by SREFLEX with the experimental data. (**D**) An overlay of the SAXS model of BlaC in Apo state with the crystal structure and SREFLEX derived model (Left), followed by two orthogonal views without the water molecules.

Convinced that there is an open-close shape change associated with activity of BlaC, we used Normal Mode Analyses (NMA) to explore the conformation space available to Apo BlaC and find a conformation which is in better agreement with the SAXS data using the program SREFLEX^29^. Briefly, the program SREFLEX works by dividing the protein into a set of pseudo-domains using either the evolutionary conservation based information or on the basis of NMA, and then running multiple cycles of generating conformers based on normal modes, discarding the conformers having severe steric clashes, ranking the remaining conformers for their agreement with the SAXS data by calculating χ^2^ and using the selected conformer for the next cycle of conformation ensemble generation. The model which conforms best to the experimental SAXS data is shown in Fig. 6A aligned with the starting crystal structure and the SAXS derived model of Apo BlaC. It can be clearly seen that the major change which leads to increased size of BlaC occurs due to the opening of the H4 helix away from the main body of the enzyme. The theoretical SAXS intensity profile of SREFLEX model is in better agreement with the experimental data with a χ^2^ value of 1.96 compared to a χ^2^ value of 3.76 for the crystal structure (Fig. 6B). The pair-wise distance distribution function of the refined model also agrees with the experimental data better compared to the crystal structure (Fig. 6C). The NMA refined model fits the SAXS derived envelope for Apo BlaC much better than the crystal structure. The extra volume observed in case of crystal structure is now occupied by a rearrangement across the H4 helix (Fig. 6D).

## Discussion

**Figure 7:**
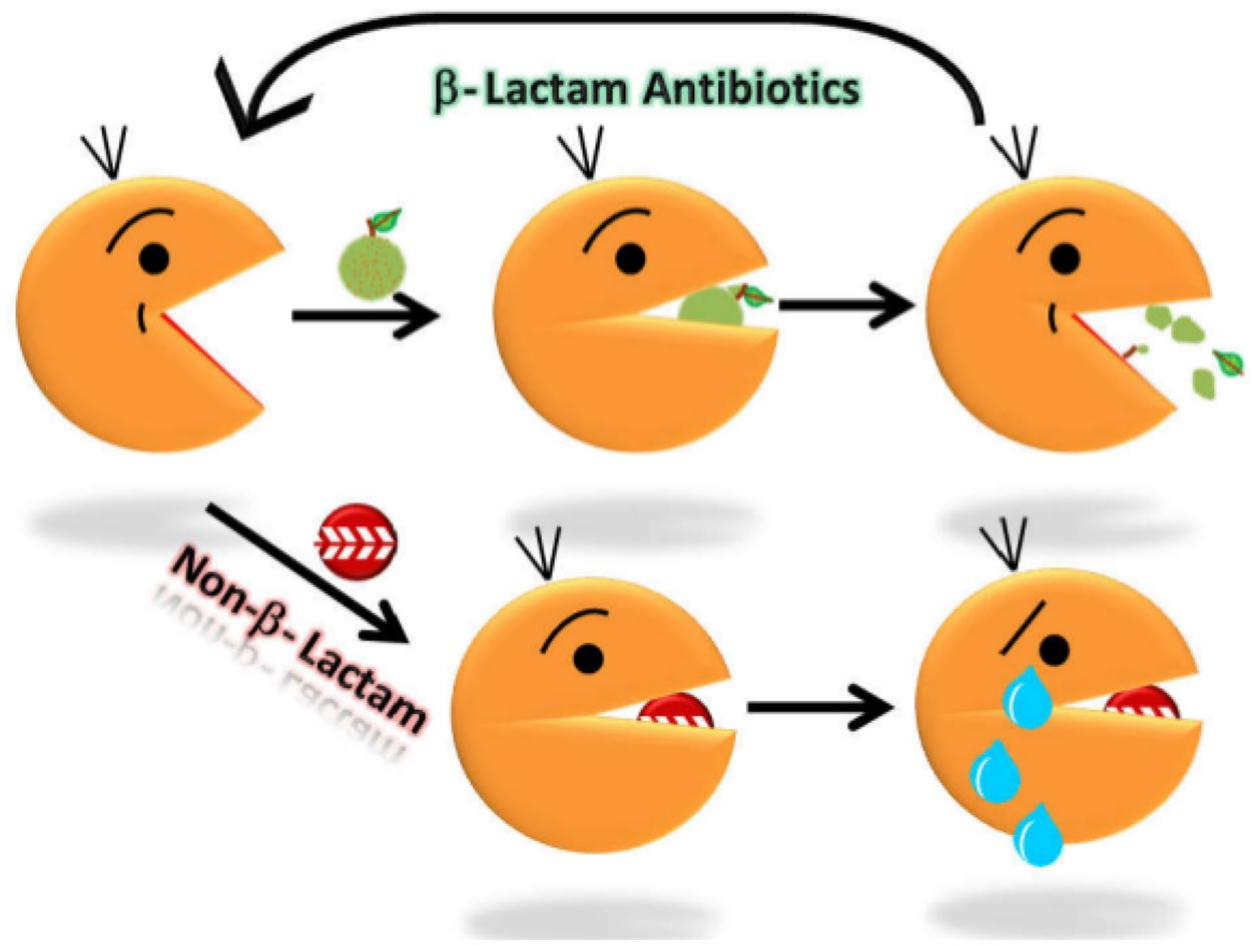
Schematic summarizing our findings that BlaC exists in a shape which has its active site open, which closes on encountering antibiotic and again opens after hydrolyzing the molecule. In contrast, a non-hydrolyzable active site binding molecule renders enzyme in closed, inactive state.

This study was initiated to search for non-lactam compounds which can tightly bind to the active site Mtb BlaC, and thus render it ineffective. In the beginning of the work, it was clear that all the known crystal structures resembled each other whether or not they were co-crystallized with inhibitor. Interestingly, even for Apo protein, the crystals were obtained with ampicillin present in the crystallization drop^7^. We wondered if there was some effect of this component during seeding of the crystals or there is only one conformation of BlaC, out of various accessible ones in solution, which has a tendency or ability to settle in a lattice which provides diffraction quality crystals? Anyway, using the crystal structure of Apo BlaC, we could find non-lactam molecules which can possibly (theoretically) fit inside and bind to the active site. Testing the lead compounds, we found four new molecules which other researchers can take forward as antibiotics against Mtb and other bugs, as such or modify them as per experimental outcome(s). One unexpected outcome of this work was the observation that solution shape of Apo BlaC was significantly larger than the crystal structures. Results described above confirm that Apo BlaC exists in open conformation, ready to accept the antibiotics in its “open” active site (Fig. 7). The data from BlaC in presence of sulbactam and tazobactam concludes that binding of antibiotic closes the enzyme, which opens again after “spewing” hydrolyzed contents. This cycle possibly repeats, until BlaC encounters a non-hydrolyzable molecule which can bind to its “open” active site viz. Clavulanate or our molecules which induces closure of mobile helix or the “lid” of the active site, but now remains closed as BlaC cannot hydrolyze them out. This renders BlaC irreversibly inactive, locked in the closed conformation. Primary outcome of our work will be of immense interest to researchers committed to structure-based drug discovery (*our NMA guided model is available on request*). Considering the ability of *Mtb* to mutate residues to escape inhibition by clavulanic acid, our molecules open up new possibilities, if not provide a proof-of-concept for others to take forward to overcome the challenge of drug induced resistance in bugs.

## Methods

### Multiple Structure Comparison

The crystal structures of BlaC solved in the presence of various inhibitors/substrates were obtained from RCSB data bank (PDB Ids: 2GDN, 3CG5, 3DWZ, 3IQA, 3M6B, 3M6H, 3N6I, 3N7W, 3N8I, 3N8R, 3N8S, 3NC8, 3NDE, 3NDG, 3NY4, 3VFF, 3VFH, 3ZHH, 4DF6, 4EBL, 4EBN, 4JLF, 4Q8I and 4X6T). The structures were aligned and compared using VMD’s MultiSeq plugin^21^. The alignment was performed using the STAMP algorithm which aligns the molecules by applying globally optimal rigid body rotations and translations in order to minimize the C^α^ distance^30^. After alignment, we found that three crystal structures *viz*. 3NBL, 3NCK and 4EBP had deletions which led to artefacts in the following calculations, so these were excluded. For the remaining aligned structures, two metrics were calculated viz. RMSD and Q_res_. RMSD, the root mean square deviation, measures the distance in angstroms between two residues in the aligned structures. Q_res_ is another measure of the similarity of protein structures^21^. It compares the C^α^-C^α^ distances between a residue and all other residues in one protein and the corresponding distances in other proteins and computes the similarity between them. The value of Q_res_ lies between 0 and 1 with higher values indicating higher similarity in the structural environment of the residue.

### In-silico Screening

The virtual ligand screening was performed on the structure of BlaC obtained in the presence of ampicillin (PDB ID: 2GDN) using the ICM 3.5 software package^31^. We docked 750,000 purchasable compounds from the ZINC database on the pocket formed by the active site. The receptor pocket was defined using ICM’s Pocket Finder module^32^. The grid maps for the defined receptor were generated which account for electrostatic potential, hydrogen-bonding, van der Waals and hydrophobic interactions. The docking simulations were run with fully flexible ligands and the docking thoroughness value of 5. The docked compounds were then sorted based on their ICM score, which was based on summation of: (1) electrostatic energy, (2) hydrophobic energy, (3) hydrogen bond interactions, (4) internal energy of the ligand, (5) reduction of the entropy of ligand upon binding and (6) hydrogen bond donor and acceptor desolvation.

### BlaC Expression and Purification

The gene for Mtb ß-lactamase was purchased from Genscript which coded for the amino acids 41–307 of BlaC. The gene was then cloned into pET28b (EMD Biosciences no. 69865-3) using the restriction sites NcoI and XhoI. This plasmid was then transformed into *Escherichia coli* BL21 (DE3) cells (EMD Bioscience no. 69387-3). As designed to aid in purification, the BlaC construct had an N-terminal 6XHis tag followed by a small stretch cleavable by thrombin. The bacteria harbouring this plasmid were grown in Luria-Bertani media having 50 µg/ml Kanamycin at 37 °C till OD_600_ reached 0.6. The target protein expression was then induced by the addition of 0.25 mM isopropyl ß-D-thiogalactopyranoside (IPTG) for 20 hours at 16 °C. The cells were pelleted by centrifugation at 6000 rpm for 20 minutes and re-suspended in the Lysis buffer containing 25 mM Tris-HCl (pH 8.0), 500 mM NaCl, and 2 mM ß-mercaptoethanol. The cells were then lysed by sonication and followed by centrifugation at 12000 rpm for 1 hour to remove the cell debris. The supernatant supplemented with 15 mM Imidazole was applied to a Ni-NTA column, followed by washing with Lysis buffer containing 25 mM Imidazole and finally, BlaC was eluted with same buffer with 250 mM Imidazole. The protein was then dialyzed against Lysis Buffer, while halving the imidazole concentration for 4 steps for 2 hours each, before completely omitting Imidazole from the dialysis buffer. Post-dialysis, the purity and approximate mass of the His-BlaC was ascertained by SDS-PAGE. This protein was subjected to thrombin (Sigma) at 18 °C for 2 hours (conditions were optimized before to get optimal results without any non-specific digestions). The tagless BlaC was purified to homogeneity by size exclusion chromatography using a Superdex S200 column attached to AKTA Prime (GE). Purity of tagless BlaC was ascertained by single band migrating in the lanes of SDS-PAGE and observation of single peak at 27.6 kDa in MALDI-TOF compared to expected value of 27.5 kDa. Protein concentrations for enzyme activity assays and SAXS experiments were estimated from observed absorbance at 280 nm using the calculated extinction coefficient value (1.06 for 1 mg/ml of protein; Protparam program at expasy.org).

### BlaC Activity Assay

The activity of BlaC was quantified using its substrate CENTA ((6*R*,7*R*)-3-[(3-Carboxy-4-nitrophenyl)sulfanylmethyl]-8-oxo-7-[(2-thiophen-2-ylacetyl)amino]-5-thia-1-azabicyclo[4.2.0]oct-2-ene-2-carboxylic acid) and recording the absorbance at 405 nm. In each reaction mixture, CENTA dissolved in DMSO was added to a concentration of 700 µM to 10 nM of BlaC. The compounds were procured from Enamine Ltd (www.enamine.net) and were added to a concentration of 200 µM to test their inhibitory potential (Supplementary Table 1). In control samples, an equal volume of DMSO was added. The absorbance at 405 nm (A_405_) was once recorded immediately after mixing the components with the measured dead time being less than 15 seconds and again after 40 minutes. Percentage inhibition (P_i_) was calculated using the following formula.

$${P}_{i}=\frac{(A{2}^{B}-A{1}^{B})-(A{2}^{C}-A{1}^{C})}{(A{2}^{B}-A{1}^{B})}\times 100$$

where, A1^B^ and A2^B^ are the first (<15 sec) and second (40 min) readings for Blank and A1^C^ and A2^C^, the corresponding readings for the tested compounds, respectively.

### SAXS Data Collection and Analyses

All SAXS data in this work was collected using SAXSpace instrument (Anton Paar GmbH, Austria). The instrument had a sealed tube X-ray source, a line collimated X-ray beam and a 1D CMOS Mythen detector (Dectris, Switzerland). The wavelength of X-rays was 0.154 nm and the sample to detector distance was 317.6 mm. Data was acquired from solutions of BlaC at concentrations of 4, 8 and 20 mg/ml to explore existence of any concentration dependent aggregation or inter-particle effect. For each concentration, the sample was exposed for 30 minutes (3 frames of 10 minutes each) at 10 °C in a thermostated quartz capillary with diameter of 1 mm. Thereafter, 100-fold molar excess of its known inhibitors clavulanic acid, sulbactam and tazobactam, and the inhibitors discovered in this study was added to BlaC and SAXS data was collected immediately for 40 minutes at intervals of 5 minutes. The data was calibrated for the beam position using SAXStreat software. The SAXSquant software was then used to subtract buffer contribution, set the usable q-range, and desmear the data using the beam profile. The SAXS data was further analyzed using the programs available in the ATSAS 2.7 suite of programs^33^. The radius of gyration (R_G_) was calculated on the basis of automated Guinier approximation using the PRIMUSQT integrated suite of programs^34^. Same suite was used to compute the distance distribution function in auto-mode using the program GNOM which performs an Indirect Fourier transformation on the SAXS intensity profile^35^. The Porod Exponent was calculated using the program Scatter3 (http://www.bioisis.net/scatter). The molecular mass of the scattering particles/protein molecules was calculated using the DATMOW program. A*b initio* models were generated using the GASBOR program^25^, and superimposed over the crystal structure and SREFLEX based model using the SUPCOMB program^26^. For modelling the Apo state of BlaC, the data collected with concentration of 20 mg/ml was used, as it had the best Signal-to-Noise ratio. Using program SREFLEX^29^, the crystal structure of BlaC (PDB ID: 2GDN) was subjected to normal mode analysis (NMA) to search for any models which may better agree with the SAXS data. The structures were visualized and the graphical representations were prepared using PyMOL v1.8^36^. The IUCr guidelines published by Jacques *et al*. were followed for reporting all the SAXS data parameters^37^.

## Electronic supplementary material

Supplementary Information
