# Supplementary material for: Search for non-lactam inhibitors of mtb β-lactamase led to its open shape in apo state: new concept for antibiotic design
Source: Sci Rep. 2017 Jul 24;7:6204. doi: 10.1038/s41598-017-06023-3 (PMC5524718; doi:10.1038/s41598-017-06023-3)

SUPPLEMENTARY INFORMATION

SEARCH FOR NON-LACTAM INHIBITORS OF Mtb β-LACTAMASE LED TO ITS OPEN SHAPE IN APO STATE: NEW CONCEPT FOR ANTIBIOTIC DESIGN

AMIN SAGAR*, NAZIA HALEEM, YAAWAR MIR BASHIR, AND ASHISH*

CSIR-INSTITUTE OF MICROBIAL TECHNOLOGY, CHANDIGARH INDIA

Supplementary Table S1

A table showing the compounds C1 through C32 which were purchased after virtual ligand screening and tested using the *In-vitro* β-lactamase activity assay. The 2D structures, IUPAC names and SMILES notations of the compounds have been tabulated with the rows colored according to the chemical moiety present in the compound. The color key is given at the bottom of the table.

	Molecule Name	Representation	IUPAC Name	SMILES
1	**C1**	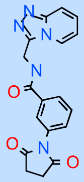	**1-(3-(((1,7,8-triaza-bicyclo[4.3.0]nona-2,4,6,8-tetraen-9-yl)-methylamino)-formyl)-phenyl)-pyrrolidine-2,5-dione**	**C1CC(N(C1=O)c1cccc(c1)C(NCc1nnc2ccccn12)=O)=O**
2	**C2**	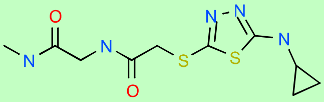	**2-(2-(5-cyclopropylamino-1,3,4-thiadiazol-2-ylsulfanyl)-acetylamino)-1-methylamino-ethanone**	**CNC(CNC(CSc1nnc(NC2CC2)s1)=O)=O**
3	**C3**	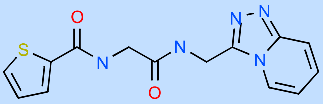	**2-((thiophen-2-yl)-formylamino)-1-((1,7,8-triaza-bicyclo[4.3.0]nona-2,4,6,8-tetraen-9-yl)-methylamino)-ethanone**	**C(C(NCc1nnc2ccccn12)=O)NC(c1cccs1)=O**
4	**C4**	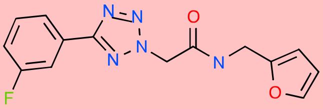	**2-(5-(3-fluoro-phenyl)-2H-1,2,3,4-tetraazol-2-yl)-1-((furan-2-yl)-methylamino)-ethanone**	**C(c1ccco1)NC(Cn1nc(c2cccc(c2)F)nn1)=O**
5	**C5**	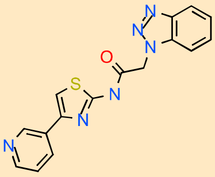	**1-(4-(pyridin-3-yl)-thiazol-2-ylamino)-2-(7,8,9-triaza-bicyclo[4.3.0]nona-1(6),2,4,8-tetraen-7-yl)-ethanone**	**C(C(Nc1nc(cs1)c1cccnc1)=O)n1c2ccccc2nn1**
6	**C6**	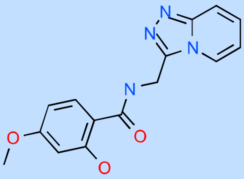	**(2-hydroxy-4-methoxy-phenyl)-((1,7,8-triaza-bicyclo[4.3.0]nona-2,4,6,8-tetraen-9-yl)-methylamino)-methanone**	**COc1ccc(C(NCc2nnc3ccccn23)=O)c(c1)O**
7	**C7**	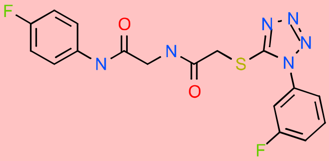	**1-(4-fluoro-phenylamino)-2-(2-(1-(3-fluoro-phenyl)-1H-1,2,3,4-tetraazol-5-ylsulfanyl)-acetylamino)-ethanone**	**C(C(Nc1ccc(cc1)F)=O)NC(CSc1nnnn1c1cccc(c1)F)=O**
8	**C8**	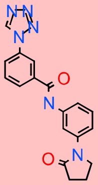	**1-(3-((3-(1H-1,2,3,4-tetraazol-1-yl)-phenyl)-formylamino)-phenyl)-pyrrolidin-2-one**	**C1CC(N(C1)c1cccc(c1)NC(c1cccc(c1)n1cnnn1)=O)=O**
9	**C9**	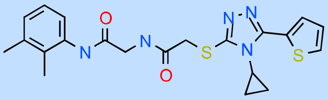	**2-(2-(4-cyclopropyl-5-(thiophen-2-yl)-4H-1,2,4-triazol-3-ylsulfanyl)-acetylamino)-1-(2,3-dimethyl-phenylamino)-ethanone**	**Cc1cccc(c1C)NC(CNC(CSc1nnc(c2cccs2)n1C1CC1)=O)=O**

	Molecule Name	Representation	IUPAC Name	SMILES
10	C10	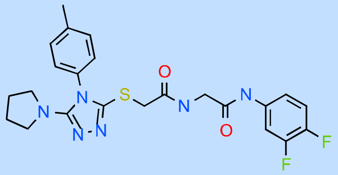	1-(3,4-difluoro-phenylamino)-2-(2-(4-p-tolyl-5-(pyrrolidin-1-yl)-4H-1,2,4-triazol-3-ylsulfanyl)-acetylamino)-ethanone	Cc1ccc(cc1)n1c(nnc1SCC(NCC(Nc1ccc(c(c1)F)F)=O)=O)N1CCCC1
11	C11	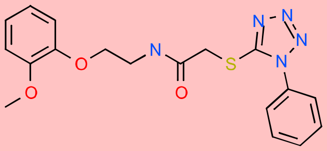	1-(2-(2-methoxy-phenoxy)-ethylamino)-2-(1-phenyl-1H-1,2,3,4-tetraazol-5-ylsulfanyl)-ethanone	COc1ccccc1OCCNC(CSc1nnnn1c1ccccc1)=O
12	C12	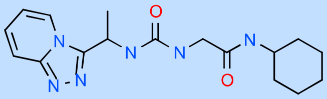	1-cyclohexylamino-2-((1-(1,7,8-triaza-bicyclo[4.3.0]nona-2,4,6,8-tetraen-9-yl)-ethylamino)-formylamino)-ethanone	CC(c1nnc2ccccn12)NC(NCC(NC1CCCCC1)=O)=O
13	C13	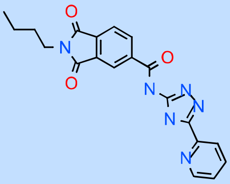	8-butyl-3-((5-(pyridin-2-yl)-4H-1,2,4-triazol-3-ylamino)-formyl)-8-aza-bicyclo[4.3.0]nona-1(6),2,4-triene-7,9-dione	CCCCN1C(c2ccc(cc2C1=O)C(Nc1nnc(c2ccccn2)[nH]1)=O)=O
14	C14	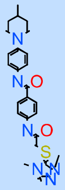	1-(4-((4-(4-methyl-piperidin-1-yl)-phenylamino)-formyl)-phenylamino)-2-(4-methyl-4H-1,2,4-triazol-3-ylsulfanyl)-ethanone	CC1CCN(CC1)c1ccc(cc1)NC(c1ccc(cc1)NC(CSc1nncn1C)=O)=O
15	C15	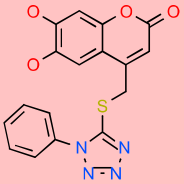	8,9-dihydroxy-3-oxo-5-((1-phenyl-1H-1,2,3,4-tetraazol-5-ylsulfanyl)-methyl)-2-oxa-bicyclo[4.4.0]deca-1(10),4,6,8-tetraene	C(C1=CC(=O)Oc2cc(c(cc12)O)O)Sc1nnnn1c1ccccc1
16	C16	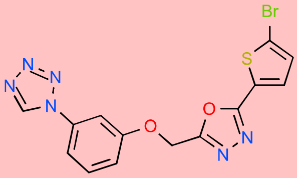	1-(3-((5-(5-bromo-thiophen-2-yl)-1,3,4-oxadiazol-2-yl)-methoxy)-phenyl)-1H-1,2,3,4-tetraazole	C(c1nnc(c2ccc(s2)[Br])o1)Oc1cccc(c1)n1cnnn1
17	C17	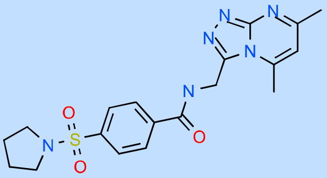	((2,4-dimethyl-1,5,7,8-tetraaza-bicyclo[4.3.0]nona-2,4,6,8-tetraen-9-yl)-methylamino)-(4-(pyrrolidin-1-ylsulfonyl)-phenyl)-methanone	Cc1cc(C)n2c(CNC(c3ccc(cc3)S(N3CCCC3)(=O)=O)=O)nnc2n1
18	C18	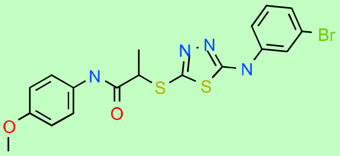	2-(5-(3-bromo-phenylamino)-1,3,4-thiadiazol-2-ylsulfanyl)-1-(4-methoxy-phenylamino)-propan-1-one	CC(C(Nc1ccc(cc1)OC)=O)Sc1nnc(Nc2cccc(c2)[Br])s1

	Molecule Name	Representation	IUPAC Name	SMILES
19	**C19**	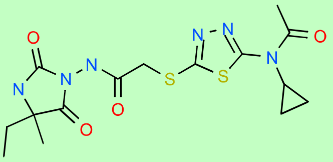	**3-(2-(5-(acetyl-cyclopropyl-amino)-1,3,4<br>-thiadiazol-2-ylsulfanyl)-acetylamino)-5-ethyl-5<br>-methyl-imidazolidine-2,4-dione**	**CCC1(C)C(N(C(N1)=O)NC(CSc1nnc(N(C2CC2)C(C)=O)<br>s1)=O)=O**
20	**C20**	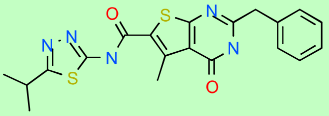	**3-benzyl-8-((5-isopropyl-1,3,4-thiadiazol-2-ylamino)<br>-formyl)-7-methyl-9-thia-2,4-diaza<br>-bicyclo[4.3.0]nona-1(6),2,7-trien-5-one**	**CC(C)c1nnc(NC(c2c(C)c3C(NC(Cc4ccccc4)=Nc3s2)=O<br>)=O)s1**
21	**C21**	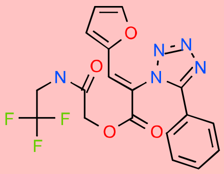	**2-oxo-2-(2,2,2-trifluoro-ethylamino)-ethyl<br>3-(furan-2-yl)-2-(5-phenyl-1H-1,2,3,4-tetraazol-1<br>-yl)-prop-2-enoate**	**C(C(F<br>)(F)F)NC(COC(C(=Cc1ccco1)n1c(c2ccccc2)nnn1)=O)<br>=O**
22	**C22**	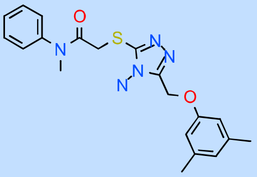	**2-(4-amino-5-((3,5-dimethyl-phenoxy)<br>-methyl)-4H-1,2,4-triazol-3-ylsulfanyl)-1-(methyl<br>-phenyl-amino)-ethanone**	**Cc1cc(C)cc(c1)OCc1nnc(n1N)SCC(N(C)c1ccccc1)=O**
23	**C23**	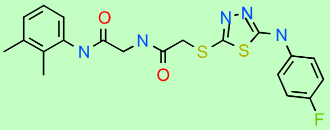	**1-(2,3-dimethyl-phenylamino)-2-(2-(5-(4-fluoro<br>-phenylamino)-1,3,4-thiadiazol-2-ylsulfanyl)<br>-acetylamino)-ethanone**	**Cc1cccc(c1C)NC(CNC(CSc1nnc(Nc2ccc(cc2)F)s1)=O)=<br>O**
24	**C24**	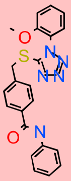	**(4-((1-(2-methoxy-phenyl)-1H-1,2,3,4-tetraazol-5<br>-ylsulfanyl)-methyl)-phenyl)-phenylamino<br>-methanone**	**COc1ccccc1n1c(nnn1)SCc1ccc(cc1)C(Nc1ccccc1)=O**
25	**C25**	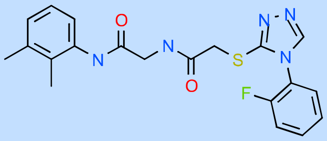	**1-(2,3-dimethyl-phenylamino)-2-(2-(4-(2-fluoro<br>-phenyl)-4H-1,2,4-triazol-3-ylsulfanyl)-acetylamino)<br>-ethanone**	**Cc1cccc(c1C)NC(CNC(CSc1nncn1c1ccccc1F)=O)=O**
26	**C26**	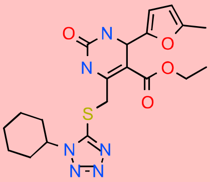	**ethyl 6-((1-cyclohexyl-1H-1,2,3,4-tetraazol-5<br>-ylsulfanyl)-methyl)-4-(5-methyl-furan-2-yl)-2<br>-oxo-1,2,3,4-tetrahydro-pyrimidine-5-carboxylate**	**CCOC(C1C(c2ccc(C)o2)NC(NC=1CSc1nnnn1C1CCCCC<br>1)=O)=O**
27	**C27**	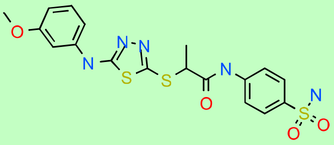	**4-(2-(5-(3-methoxy-phenylamino)-1,3,4<br>-thiadiazol-2-ylsulfanyl)-propionylamino)<br>-benzenesulfonamide**	**CC(C(Nc1ccc(cc1)S(N<br>)(=O)=O)=O)Sc1nnc(Nc2cccc(c2)OC)s1**

	Molecule Name	Representation	IUPAC Name	SMILES
28	**C28**	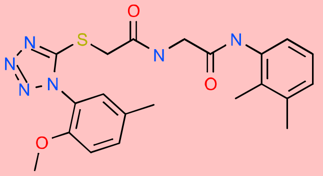	**1-(2,3-dimethyl-phenylamino)-2-(2-(1-(2-methoxy-5-methyl-phenyl)-1H-1,2,3,4-tetraazol-5-ylsulfanyl)-acetylamino)-ethanone**	**Cc1ccc(c(c1)n1c(nnn1)SCC(NCC(Nc1cccc(C)c1C)=O)=O)OC**
29	**C29**	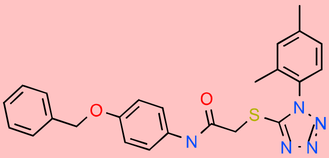	**2-(1-(2,4-dimethyl-phenyl)-1H-1,2,3,4-tetraazol-5-ylsulfanyl)-1-(4-(phenyl-methoxy)-phenylamino)-ethanone**	**Cc1ccc(c(C)c1)n1c(nnn1)SCC(Nc1ccc(cc1)OCc1ccccc1)=O**
30	**C30**	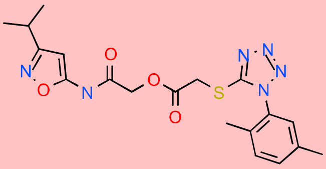	**2-(3-isopropyl-isoxazol-5-ylamino)-2-oxo-ethyl 2-(1-(2,5-dimethyl-phenyl)-1H-1,2,3,4-tetraazol-5-ylsulfanyl)-ethanoate**	**CC(C)c1cc(NC(COC(CSc2nnnn2c2cc(C)ccc2C)=O)=O)on1**
31	**C31**	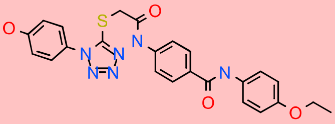	**1-(4-((4-ethoxy-phenylamino)-formyl)-phenylamino)-2-(1-(4-hydroxy-phenyl)-1H-1,2,3,4-tetraazol-5-ylsulfanyl)-ethanone**	**CCOc1ccc(cc1)NC(c1ccc(cc1)NC(CSc1nnnn1c1ccc(cc1)O)=O)=O**
32	**C32**	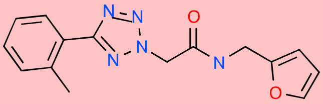	**1-((furan-2-yl)-methylamino)-2-(5-o-tolyl-2H-1,2,3,4-tetraazol-2-yl)-ethanone**	**Cc1ccccc1c1nnn(CC(NCc2ccco2)=O)n1**

Compounds having Tetrazole moiety

Compounds having 1,3,4 - Triazole moiety independently or as part of a larger ring

Compounds having Thiadiazole moiety independently or as part of a larger ring

Compounds having 1,2,3 - Triazole moiety independently or as part of a larger ring

Data-collection and scattering-derived parameters

Supplementary Table 2

**Data-collection parameters**	
Instrument /	SAXSpace (Anton Paar)
Beam geometry	10 mm slit
Wavelength (Å)	1.5418
Desmearing Software	Done using SAXSquant
q range (nm^{-1})	0.10–3.00
Temperature (K)	283
**Molecular-mass determination†**	
Partial specific volume (cm^3 g^{-1})	0.724
Calculated monomeric M_r from sequence	27.5
Dry volume calculated from sequence ($Å^3$)	34190
**Software employed**	
Primary data reduction	*SAXSquant*
Data processing	*PRIMUS QT*
Ab initio analysis	*GASBOR*
Validation and averaging	*DAMAVER*
Rigid-body modeling	N/A
Computation of model intensities	*CRYSOL*
Three-dimensional graphics representations	*PyMOL*

Table 2A

	**Apo BlaC (Conc. Series)**	**Apo BlaC (Conc. Series)**	**Apo BlaC (Conc. Series)**
**Data-collection parameters**			
Exposure time (min)	30	30	30
Concentration range (mg ml^{-1})	20	8	4
I(0)/C	886.1	880.0	888.3
**Structural parameters†**			
I(0) (Arbitrary Units) [from *P(r)*]	17820± 201	70±132	3485±104
R_g (nm) [from *P(r)*]	2.27± 0.04	2.30 ± 0.09	2.45 ± 0.16
I(0) (Arbitrary Units) (from Guinier)	17722± 284	7040±147	3553±110
R_g (nm) (from Guinier)	2.35 ± 0.012	2.38 ± 0.018	2.30 ± 0.025
D_{max} (nm)	6.7	6.8	6.9
Porod volume estimate ($Å^3$)	38503	38258	38365
**Molecular-mass determination**			
Molecular mass M_r [from V_c]	29.1	29.3	29.4

Table 2B

	Apo BlaC (Time Series)	BlaC+ CA (Time Series)	BlaC+ SB (Time Series)	BlaC+ TB (Time Series)	BlaC+ C5 (Time Series)	BlaC+ C13 (Time Series)	BlaC+ C16 (Time Series)	BlaC+ C28 (Time Series)
**Data-collection parameters**								
Exposure time (min)	5	5	5	5	5	5	5	5
Concentration range (mg ml^{-1})	20	20	20	20	20	20	20	20
**Structural parameters†**								
I(0)<br>(from Guinier)	2968.24±135.43<br>2940.64±134.78<br>2911.41±139.99<br>2965.41±158.33<br>2934.97±136.12<br>2996.96±150.07<br>2966.75±161.12<br>2980.64±149.24	2958.99±151.07<br>2921.74±145.66<br>2969.78±150.96<br>2912.41±146.74<br>2955.25±145.32<br>2895.58±142.13<br>2958.35±148.42<br>2916.95±139.67	2914.08±142.78<br>2928.80±145.20<br>2953.57±150.79<br>2926.70±146.45<br>2937.87±150.68<br>2916.62±143.17<br>2941.73±151.52<br>2914.19±144.59	2940.78±143.75<br>2915.13±146.55<br>2996.80±147.41<br>2986.03±152.93<br>2980.35±149.17<br>2949.37±144.91<br>3024.01±156.85<br>2986.82±147.19	2932.99±145.29<br>2895.24±144.48<br>2954.58±146.53<br>2955.09±151.13<br>2930.92±143.42<br>2939.73±144.05<br>2973.23±146.34<br>2916.58±143.18	2951.25±150.28<br>2917.09±147.83<br>2997.49±146.42<br>2964.95±152.73<br>2926.70±147.20<br>2967.04±151.36<br>2931.05±145.34<br>2956.11±148.36	2940.90±147.00<br>2934.89±150.07<br>2956.45±154.29<br>2939.49±152.14<br>2951.54±146.20<br>2953.19±151.78<br>2969.84±147.45<br>2973.35±148.37	2908.22±145.29<br>2950.68±151.43<br>2962.11±146.44<br>3010.84±147.61<br>2922.80±149.83<br>2909.93±141.36<br>2975.66±143.54<br>2985.02±150.76
R_g(nm)<br>(from Guinier)	2.35±0.048<br>2.32±0.053<br>2.33±0.058<br>2.31±0.047<br>2.34±0.060<br>2.32±0.042<br>2.36±0.031<br>2.34±0.040	2.01±0.041<br>1.98±0.048<br>1.97±0.05<br>2.01±0.038<br>2.04±0.042<br>2.03±0.036<br>2.04±0.033<br>2.04±0.04	1.97±0.035<br>2.04±0.031<br>2.15±0.039<br>2.28±0.041<br>2.34±0.042<br>2.36±0.034<br>2.32±0.036<br>2.34±0.041	1.98±0.029<br>2.09±0.038<br>2.13±0.044<br>2.16±0.031<br>2.34±0.027<br>2.32±0.033<br>2.31±0.028<br>2.30±0.030	1.98 ± 0.03<br>2.02 ± 0.039<br>2.04 ± 0.06<br>2.10 ± 0.046<br>2.05 ± 0.051<br>2.12 ± 0.041<br>2.13 ± 0.039<br>2.09 ± 0.034	2.04 ± 0.033<br>1.94 ± 0.047<br>2.09 ± 0.029<br>2.08 ± 0.066<br>2.05 ± 0.054<br>2.09 ± 0.032<br>2.11 ± 0.040<br>2.04 ± 0.038	1.98 ± 0.048<br>1.95 ± 0.041<br>2.06 ± 0.039<br>2.04 ± 0.052<br>2.10 ± 0.032<br>2.12 ± 0.037<br>2.07 ± 0.044<br>2.09 ± 0.030	2.11 ± 0.054<br>2.06 ± 0.032<br>2.13 ± 0.047<br>2.09 ± 0.039<br>2.13 ± 0.042<br>2.14 ± 0.034<br>2.08 ± 0.043<br>2.09 ± 0.033
Porod volume estimate ($Å^3$)	38091<br>37667<br>38358<br>39073<br>38012<br>38937<br>39154<br>38460	37840<br>39001<br>38137<br>38387<br>38346<br>37441<br>38540<br>38639	38912<br>38448<br>38827<br>38650<br>38182<br>38156<br>38568<br>37693	38119<br>38340<br>38912<br>38518<br>39001<br>38153<br>39085<br>38356	37947<br>38606<br>38189<br>38973<br>38523<br>38171<br>39235<br>38225	38325<br>38466<br>38346<br>38946<br>38093<br>38117<br>38199<br>38156	38429<br>39052<br>39079<br>39010<br>38485<br>38951<br>38620<br>38577	38925<br>38645<br>38320<br>39254<br>38454<br>38985<br>38092<br>38463
**Molecular-mass determination**								
Molecular mass M_r [from V_c]	28.9<br>28.8<br>29.5<br>28.7<br>28.6<br>29.2	29.2<br>28.7<br>29.1<br>29.0<br>28.9<br>29.0	29.2<br>29.4<br>29.4<br>28.8<br>29.9<br>29.2	29.0<br>29.1<br>28.9<br>28.9<br>29.5<br>28.9	29.3<br>29.1<br>29.0<br>28.6<br>28.8<br>29.5	29.3<br>28.9<br>29.3<br>29.2<br>28.9<br>29.4	29.1<br>28.6<br>29.3<br>29.0<br>28.5<br>28.8	29.1<br>28.9<br>28.8<br>28.9<br>29.2<br>28.7

Supplementary Figure Legends

Figure S1 - The SAXS intensity profiles of BlaC in Apo state (A) and in presence of 100 fold molar excess of Clavulanic acid (B), Sulbactam (C), Tazobactam (D), C5 (E), C13 (F), C16 (G) and C28 (H) at various time points from 5 to 40 min.

Figure S2 - The Guinier Plots of BlaC in Apo state (A) and in presence of 100 fold molar excess of Clavulanic acid (B), Sulbactam (C), Tazobactam (D), C5 (E), C13 (F), C16 (G) and C28 (H) at various time points from 5 to 40 min. In the inset of each plot, the linear fits of the Guinier Plots at 5 and 40 min are presented close to each other to clearly show the difference in the slopes.

Figure S3 - The Porod-Debye Plots of BlaC in Apo state (A) and in presence of 100 fold molar excess of Clavulanic acid (B), Sulbactam (C), Tazobactam (D), C5 (E), C13 (F), C16 (G) and C28 (H) at various time points from 5 to 40 min. The gray histograms represent the value of Porod Exponent calculated from the SAXS profile.

Figure S4 - The Normalized Kratky Plots of BlaC in Apo state (A) and in presence of 100 fold molar excess of Clavulanic acid (B), Sulbactam (C), Tazobactam (D), C5 (E), C13 (F), C16 (G) and C28 (H) at various time points from 5 to 40 min.

Supplementary figure S1

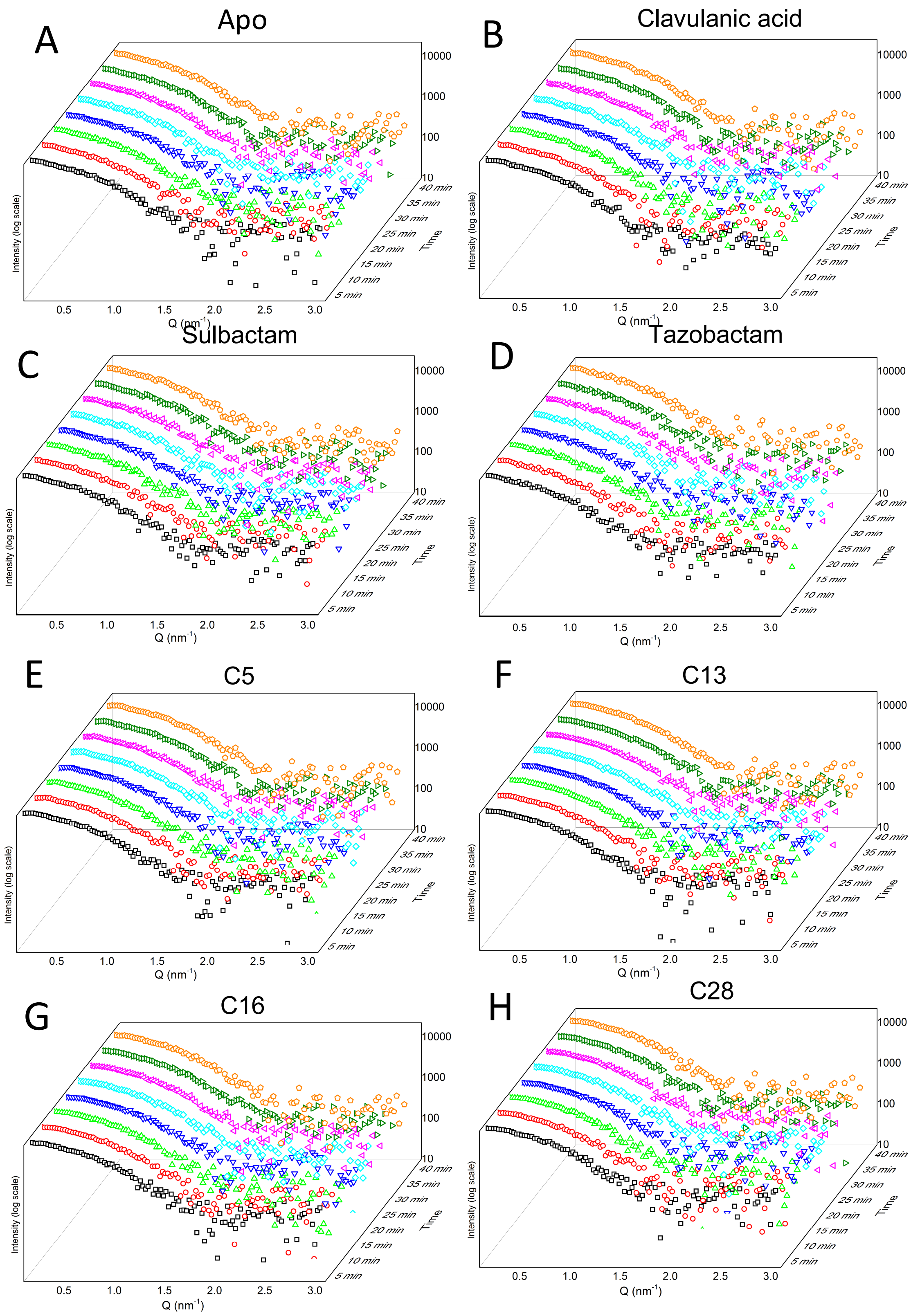

Supplementary figure S2

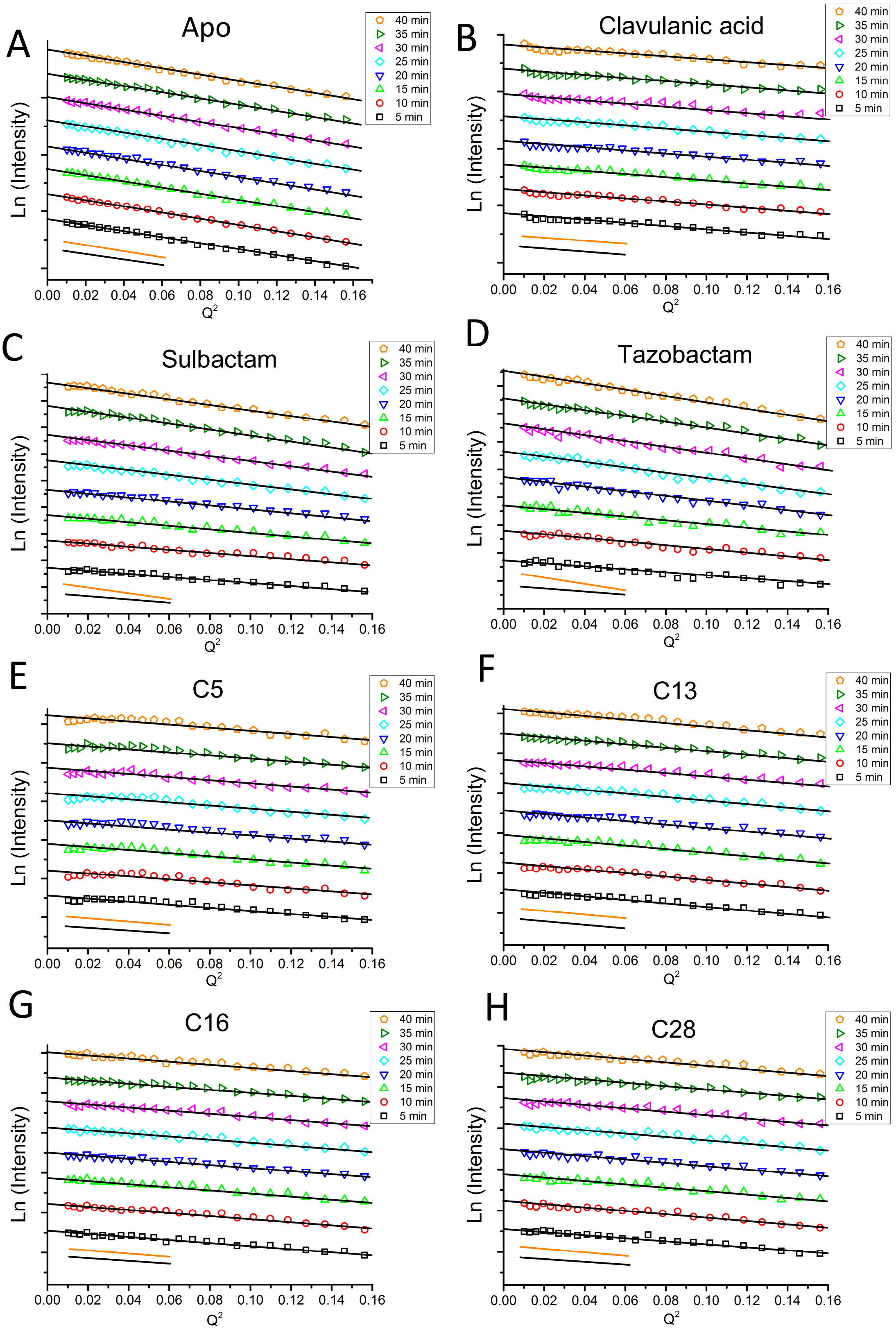

Supplementary figure S3

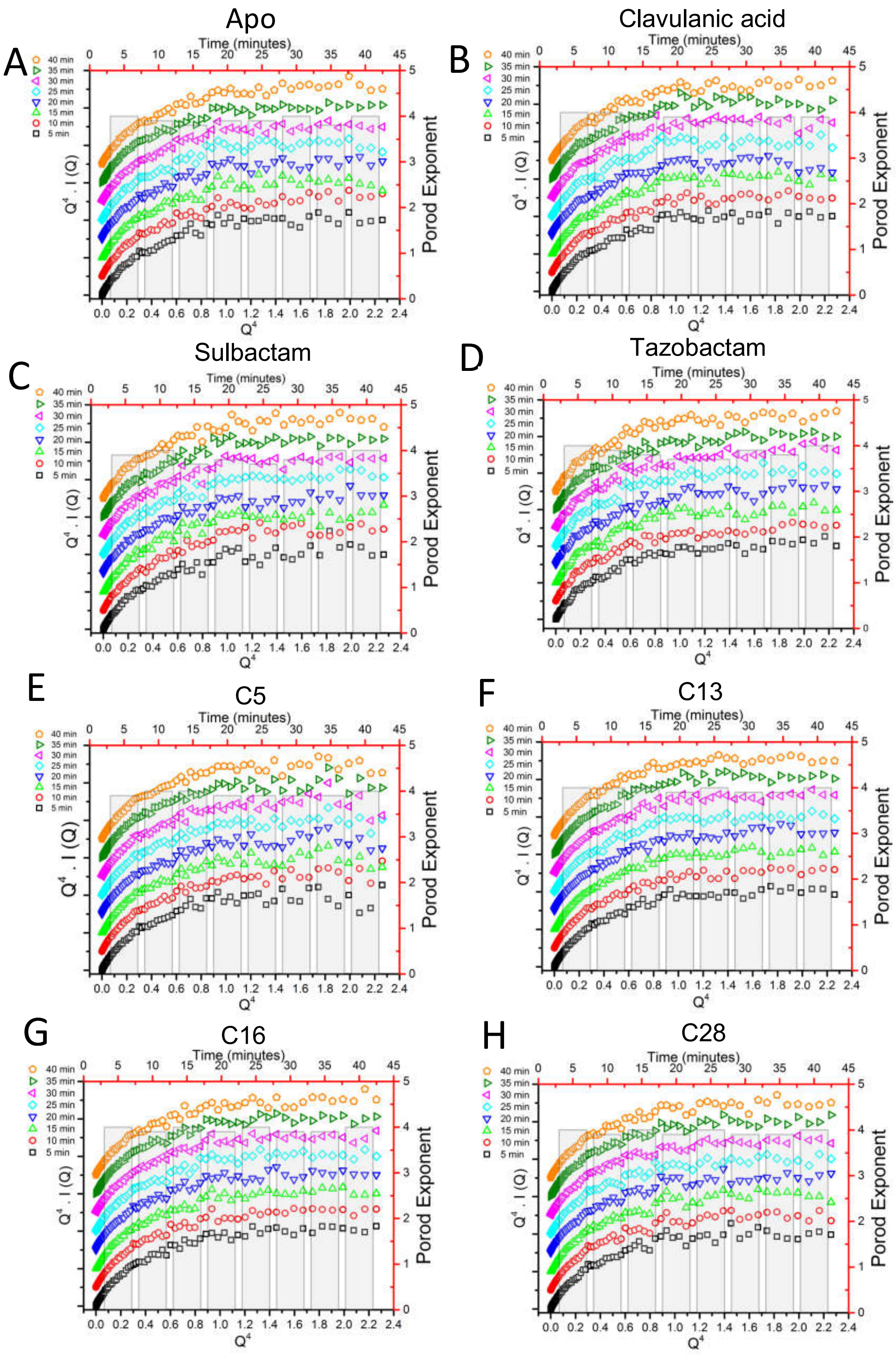

Supplementary figure S4

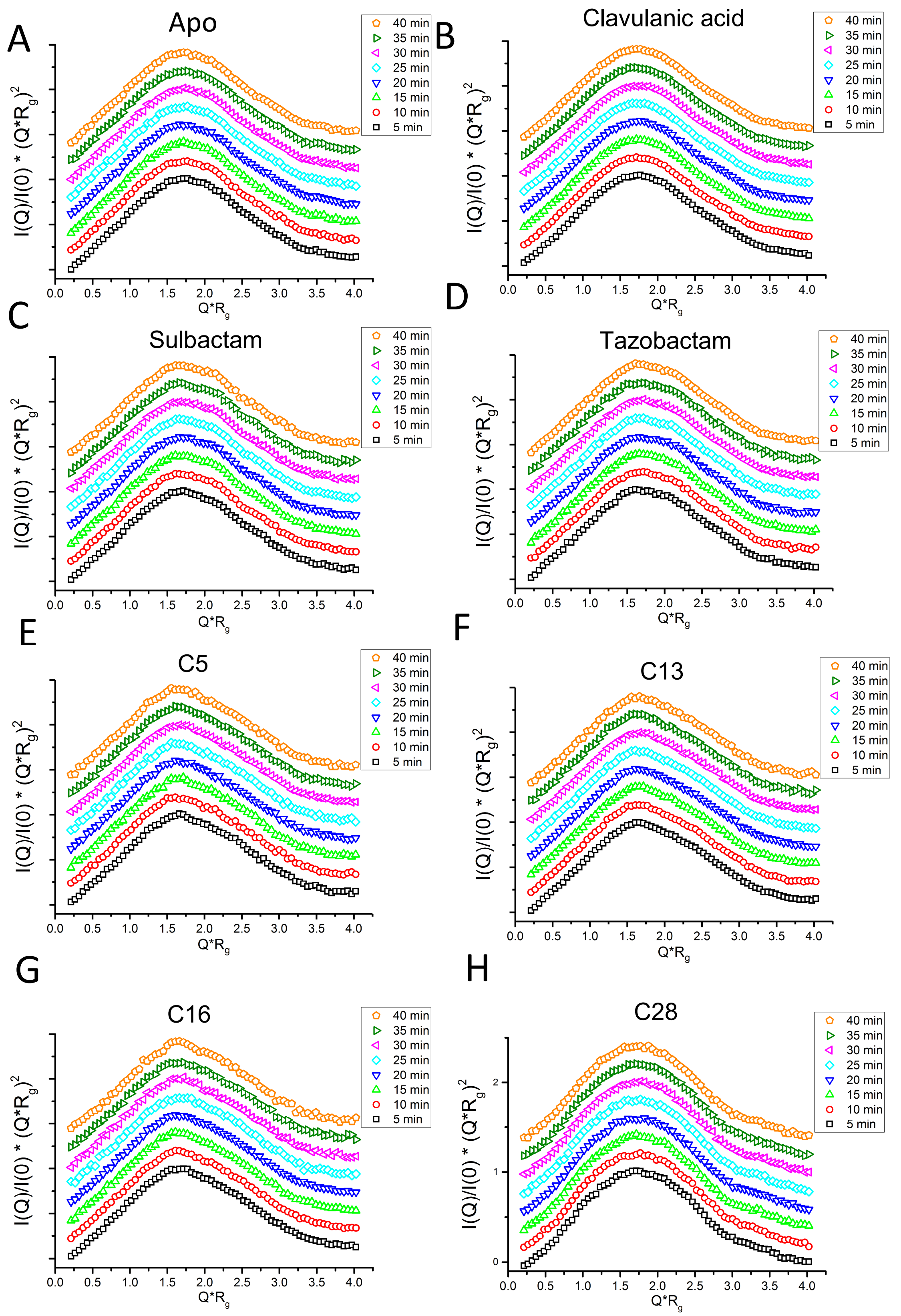

Supplement: Supplementary file 1 — Supplementary Information [file 41598_2017_6023_MOESM1_ESM.pdf]
